# Power Converter Design for Pulsed Electric Field-Based Milk Processing: A Proof of Concept

**DOI:** 10.3390/foods14132177

**Published:** 2025-06-21

**Authors:** Julieta Domínguez-Soberanes, Omar F. Ruiz-Martinez, Fernando Davalos Hernandez

**Affiliations:** Facultad de Ingeniería, Universidad Panamericana, Josemaría Escrivá de Balaguer, 101, Villa Bonaterra, Aguascalientes 20296, Mexico; jdominguez@up.edu.mx

**Keywords:** pulsed electric field, dairy foods, novelty, food non-thermal process, food quality, power electronic converter

## Abstract

The microbiological safety of milk can be ensured through heat processing; however, this method has a negative effect on the sensory profile of this food product. Emerging technologies could be used as an alternative process for guaranteeing innocuity and maintaining sensory changes. An alternative is to evaluate pulsed electric field (PEF) electroporation, which is a method of processing cells using short pulses of a strong electric field. PEF has the potential to be a type of alternative low-temperature pasteurization process that consists of high-frequency voltage pulsations. Specifically, the presented work is a proof of concept for the design of a converter capable of generating a PEF to feed a load that meets the impedance characteristics of milk. The proposed converter is simulated using PLECS software (4.9.6 version) under impedance change scenarios that emulate variations in milk throughout the entire process. This research proposes the modification of a classic Vienna rectifier (adding an MBC—Multilevel Boost Converter structure) to supply a pulsating signal that could be used for low-temperature processes of milk to guarantee proper pasteurization. The characteristics of the generated high-voltage pulse make it feasible to quickly process the real sample. The control law design considers a regulation loop to achieve a voltage in the range of kV and a switching-type control law that activates switches in MMC arrays. These switches are activated randomly to avoid transients that cause significant stress on them.

## 1. Introduction

Milk is a biologically complex fluid that contains all macromolecules such as proteins, carbohydrates, lipids, minerals, and vitamins. In addition to this nutritional load, dairy products are a source of bioactive peptides, prebiotics and probiotics, and essential fatty acids such as CLA [1,2].

Moreover, it is known that this food product has a unique composition, making it easily contaminated due to the richness of its food components. Its richness makes it an excellent medium for microorganisms to grow. Therefore, it is important to highlight that food safety in these types of products has been an issue over the last few years. Raw milk has been associated with pathogens such as *Listeria monocytogenes*, *Staphylococcus aureus*, *Escherichia coli* O157:H7, *Bacillus cereus*, *Salmonella* spp., *Campylobacter* spp., and *Clostridium botulinum*, which have led to diseases and death [3].

As a result, heat treatments have been applied to guarantee consumer safety. These treatments reduce the number of pathogenic microorganisms present in the food product. However, this process can lead to physical and chemical changes in protein structure and functionality, having as a side effect the destruction of nutritional components in this food product, which, as a consequence, affects the sensory characteristics of fluid milk, changing its flavor and odor [3,4,5]. The most commonly used/reported heat treatments in the milk industry are described below.

1. Thermalization: This method heats milk at 60–69 °C for 20–30 s [5] or at 57–68 °C for 5 s up to 30 min, causing the death of non-heat-resistant bacteria and inactivating several enzymes [5,6,7].
2. Pasteurization: Both pasteurization processes cause the death of most pathogens, vegetative bacteria, yeasts, and molds. Moreover, they cause the denaturation of several enzymes and several whey proteins [5]. They inactivate non-spore-forming pathogens, psychrotrophic spoilage bacteria, and thermoducric bateria [5,6,7].
  - Low-pasteurization long-time (LTLT) pasteurization: This heats milk at 63–65 °C for 30 min. This process is a regulatory requirement for the pasteurization of raw milk [5,6,7].
  - High-temperature short-time (HTST) pasteurization: 72–75 °C for 15–20 s [5,6,7].
3. High Pasteurization: This method heats milk at 85 °C for 20–30 min or applies heat at 90–95 °C for 5 min and results in the death of most vegetative microorganisms, except spores. It deactivates most enzymes, denatures most whey proteins, and develops flavors that are not well accepted [5,6,7].
4. Sterilization: This method heats milk at 110 °C for 30 min or at 130 °C for 40 s, which results in the extermination of all microorganisms, deactivation of most enzymes, denaturation of whey proteins, and aggregation of caseins (*casein micelles*), but it affects the sensory characteristics of the product, leading to weakened flavor intensity and color darkening [5,6,7].
5. Ultra heat treatment (UHT): This method heats milk at 145 °C for 1–2 s and causes extermination of all microorganisms, mild flavor deterioration, denaturation of whey proteins, and development of off-flavors and darkening [5,6,7].

The above information is displayed in Table 1 to clarify the characteristics of each process.

Due to effects on the sensory properties of milk that have been observed in thermal processes, there has been a growing global demand to develop minimally processed milk. The proposed methods are a combination of both non-thermal and thermal technologies (with mild intensity (time and temperature) and/or only using a non-thermal technology).

Novel non-thermal technologies have emerged as an alternative, taking into account that the challenge is to inactivate both pathogenic and spoilage microorganisms. These methods include the following: high-pressure processing (HPP), pulsed electric field (PEF), ultrasound, ultraviolet irradiation, non-thermal plasma (cold plasma), and membrane microfiltration [3,8,9].

Pulsed electric field (PEF) technology could have important industrial applications in the dairy industry. It has been established that PEF processing in liquid products can guarantee microbial safety under certain conditions.

(I) The most important variables influencing the characteristics of the food medium that we have to consider include the following:

1. Microorganisms contained in food:
  - Type;
  - Size;
  - Cell surface structure;
  - Concentration growth phase.
2. Ionic strength;
3. pH;
4. Water activity;
5. Viscosity;
6. Presence of solid particles or oil droplets.

It is known that the inactivation of microorganisms is more effective when the medium has high electric resistivity. On the other hand, it is known that increases in the concentration of protein and lipids appear to increase microbial resistance to electric pulses [9,10].

(II) Process parameters. Three characteristics have to be considered:

1. Field strength;
2. Treatment time;
3. Intensity.

For example, The results presented in [10] established that high voltage (10–50 kV cm^−1^) and very short electric pulses (less than 10 µs) could pasteurize certain types of fluid foods [9,10]. Other ways of processing milk have been proposed in camel milk, such as high-pressure processing, pulsed electric fields, ultrasonic processing, UV pasteurization, cold plasma technology, and microwave processing. Some of these approaches offer benefits to nutrient retention, improved sensory analysis, and food safety [11] Therefore, emerging technologies that enhance food safety and quality and prolong shelf life by eliminating microbe contamination with minimal nutritional loss and sensory characteristics are needed [5].

One promising technology that has emerged to process food more efficiently and avoid spoilage is the irradiation of food with a pulsed electrical field [12,13,14,15,16]. This constitutes a type of electroporation that helps cause a significant increase in electrical conductivity and permeability of the plasma membrane, and once the applied voltage exceeds the dielectric strength of the membrane, pores are formed [17,18]. It has been established that this method has multiple applications in various areas such as the following: improving the production or extraction of certain compounds in plants [19,20], increasing the properties of certain foods [21], sterilization of liquids [22], treatment of cells for disease analysis purposes [23,24,25], etc. Diffusion processes, such as the removal of water from plant or animal tissues (for a dehydration process) or the absorption of marinades, spices, and auxiliary substances, are accelerated, thus reducing the time in production processes. Other advantages attributed to PEF (pulsed electric field) include improved extraction rates of juices, sugars, and other active substances, as well as significantly extended food preservation.

The realization of the PEF process is through the design of a chamber, where the food is housed, and by means of electrodes, a pulsating voltage signal of a few kilovolts in amplitude is irradiated for a few microseconds or milliseconds [26,27,28]. The design of the equipment that develops this technology is based on the control of power converters that are oriented towards voltage regulation to feed a load despite disturbances. The literature is extensive regarding the presentation of various types of converters in all forms of voltage transformation (DC-DC, DC-AC, AC-AC, AC-DC). In order to provide a pulsating voltage signal, it is necessary to modify the operation of the converter or its structure to ensure that the voltage amplitude is constant and associated with a certain frequency.

In general, the type of converter used to provide a PEF signal is a CD-CD type. For this converter to behave as a high-voltage pulse generator, the principle of storing/charging energy in capacitors or inductors and then abruptly discharging the energy through the load is used. It is reported in the literature that various charging stages with diode–capacitor or diode–inductor arrangements are used to achieve very high voltage levels [27,29]. This way of achieving high voltages helps avoid the use of switches directly connected to the output, which would have to withstand and block large current and voltage transients through their structure [30]. Although these reported converter topologies exhibit high efficiency and ease of control, they present the disadvantage of requiring a previous stage that provides a high DC voltage (another converter, some set of batteries, several DC sources, or a high-voltage source) [31]. That is, in general, these systems are made up of two stages, and this leads to an additional cost to that contemplated in the design of the converter that generates the PEF. Additionally, the power required to produce the PEF depends on the capacity of the supply source and not the converter.

In general, the limitations in the performance of this type of converters (PEF generators) are because they require a DC voltage source at the input that supports the demanded power, and additionally, the frequency of the high-voltage pulses depends on the characteristics of passive networks (charge–discharge time τ). For example, in [32], a Boost structure circuit is presented with a DC source as input with a Blumlein arrangement that generates low-voltage unipolar pulses (500V), whose pulse duration depends on the parameters R and L, which also define whether the converter operates in continuous or discontinuous conduction mode.

In [33], a DC-powered converter is presented and requires connecting multiple switch modules in series and parallel to obtain high-voltage bipolar pulses (<10 kV) at its output. The number of switches per module increases the control complexity due to the synchronization that the switching signals must show. In [34], a topology for bipolar pulses based on MMC arrangements similar to our proposal is presented, but it requires a DC source that limits the power capacity, and for certain effects, it requires a transistor at its output that must support/block the high voltage levels (10 kV) generated.

In [35], a converter powered by a battery bank is presented that can generate unipolar pulses but produces low-frequency pulses due to the use of thyristors to handle high power. Reference [36] presents a structure also based on MMC with a simple PI control loop that allows generating high-frequency pulses at high voltage (5 kV), but its power is limited by the input DC source. The way to obtain bipolar pulses is through the inclusion of a Dual Boost converter, but the disadvantage is that the input switches are subjected to significant stress.

In [37], a structure is presented that uses the current multiplication principle of MARX arrays but uses high-frequency transformers. This arrangement has the advantage of using the transformation ratio to obtain very-high-voltage pulses. A disadvantage is that the switches are in series with the primaries of each transformer, and this means that they must support a considerable current (100 A), and additionally, the pulse duration is limited to a few milliseconds with an approximate exponential decay.

In [38], a topology based on boost converter arrays (with DC source supply) is presented that induces a current in the primary of transformers with their coupled secondaries to obtain a high voltage (10 kV), but the pulses obtained are unipolar. The pulses are obtained through MMC structures similar to the one used in the present proposal. In [39], a structure based on an input half bridge with transformer coupling (forming an LLC array) is proposed to boost the voltage. The pulses obtained are high-voltage (1 kV), unipolar, and sawtooth-shaped due to the transformer effect. The pulse duration is on the order of milliseconds.

The structure of modified conventional converters has been explored in [17,18], where topologies using RLC structures are presented. The resonant effect causes a unipolar type of pulsed signal. The switches in these structures are required to withstand/handle very high voltages (around 50% of the desired output voltage).

Topologies based on bridge-type circuits are proposed in [22,31] to generate high-voltage bipolar pulses (1–2 kV) with pulse durations of some microseconds. The disadvantage is that they do not have a boost stage and require the connection of multiple input sources to reach a high voltage. The bridge-type structure is designed to direct a significant current in a bidirectional manner. In contrast to what was described above, our proposal presents a converter that does not require a high-power DC source to operate, the Vienna rectifier structure requires simple capacitor modifications to significantly increase the voltage level (MBC structure), the output pulses are bipolar and are made through MMC structures that avoid significant stress on the switches, and finally, the pulse durations are uniform and on the order of microseconds.

Therefore, the objective of this paper is to present a proof of concept for a power converter design intended for use in raw milk processing. As stated, the PEF method generates a high-voltage, high-frequency pulsating electric field that is applied to the food for a short duration, typically measured in milliseconds. The most outstanding advantage of this process is that it avoids the typical heating in pasteurization to eliminate bacteria. Moreover, this work presents the operation and design of the proposal through simulations without considering the modeling of the treatment chamber.

The table below (Table 2) shows a summary of the key features of the pulse generators described above in comparison with our proposal.

The article is organized in the following manner: In Section 2, the basic concepts and principles to take into account are developed. In Section 3, the converter operation is developed and explained in conjunction with the design of the switching law. In Section 4, the simulation results are presented, and finally, in Section 5, the final conclusions are outlined together with possible improvements to be developed as future work.

## 2. Mechanism of PEF and Power Converter Basis

There are two ways to treat foods using PEF. One is by applying the PEF to an already packaged product and the other is by applying it to the product in its natural state prior to being packaged [16]. In this work, the second case is explored.

**Figure 1:**
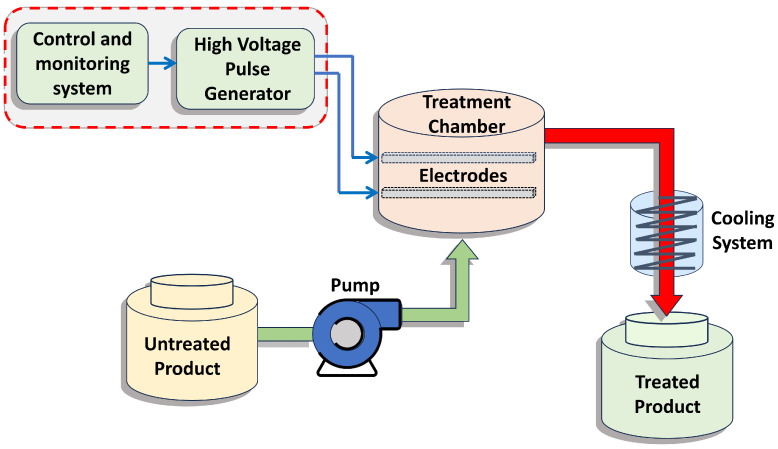
Elements for the PEF-based process.

Figure 1 shows the complete PEF-based process for liquid samples consisting of three basic elements: a high-voltage pulse generator (converter), an isolated treatment chamber, and a process control and monitoring system. The tanks are designed to provide a storage system before and after treatment, and the pump provides a flow at a constant speed to standardize the treatment time. The circuit together with a controller allows defining the signal operating intervals and its amplitude. The generation of the pulsating electric field requires a fast discharge of energy in short periods of time. This is generally achieved by charging and discharging capacitors through a switch configuration to a load configured in this case as electrodes within the treatment chamber.

The strength of the electric field is one of the important parameters to control, but the way in which the electric field is produced must also be controlled by determining the number of pulses in a given period (*n*), the duration or width of the pulse ($t_{p}$), the frequency, and the dynamics in which it is generated. The treatment time through the PEF ($T_{PEF}$) is defined as the duration (in μS) of the pulse multiplied by the number of pulses applied in a given period, which is as follows: $T_{PEF}=t_{p}\times n$.

**Figure 2:**
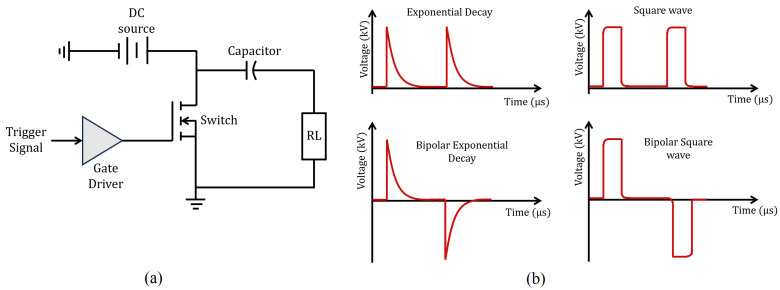
Pulsed waveforms and the basic circuit to generate them: (**a**) Basic process to generate the voltage pulses. (**b**) Different pulse waveforms.

Figure 2a shows the basic way of generating voltage pulses at a given frequency. This is achieved through a switch that connects and disconnects a voltage source that feeds a capacitor. The capacitor is charged and discharged at regular intervals through resistive loading equivalent to the impedance displayed by the electrodes within the treatment chamber. As can be seen, the limitation in generating high-voltage pulses depends on the capacity of the source, and the operating frequency depends on the signal that activates the switch. Modern converters are configured by combinations of switches and passive elements to achieve the appropriate voltage levels. Once an appropriate operation has been defined for the converter configuration, it is necessary to design and define switching signals for each switch and thus generate high-voltage pulses, that is, design a control law. This control law will allow achieving a pulsating behavior at the output that can have different characteristics depending on the type of electric field to be generated within the treatment chamber.

Figure 2b shows the most common ways to generate electrical pulses, namely exponential decay and square waveforms [18,29]. Square waveforms are considered ideal for these applications because they present uniformity throughout the pulse duration. Note that the signal must be of a magnitude of kV with alternating polarity. Some power converter topologies can deliver very high voltages, but due to their nature, they deliver regulated voltages without variations. Before moving on to the proposal development, the basic principles of converter operation and the basic design of a switched control law are presented. This is preliminary information to establish the characteristics that subsequent development should follow.

Before proceeding to explain the theoretical design of the converter valid for the PEF, it is advisable to make some clarifications about restrictions and operating characteristics.

**Figure 3:**
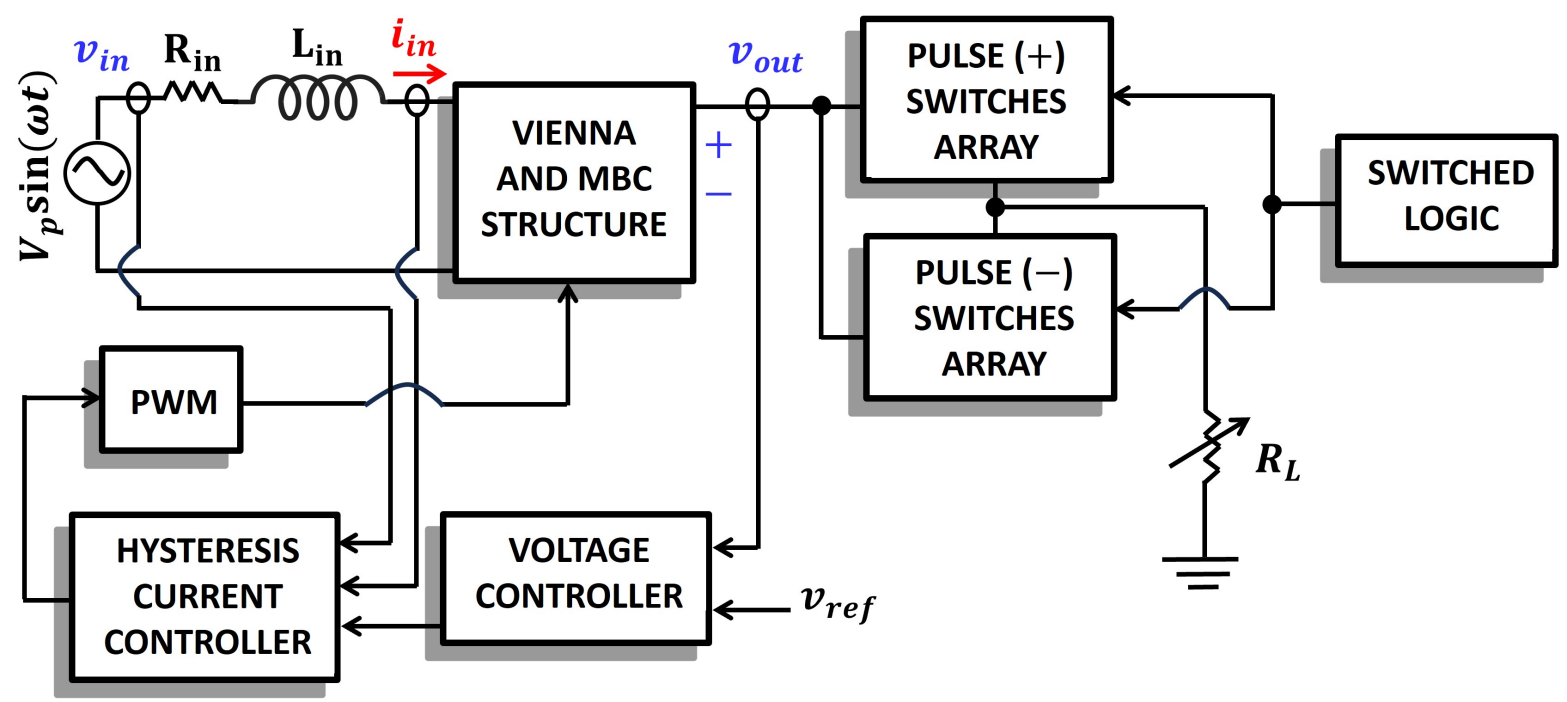
Basic Block Diagram of the proposed converter.

1. As can be seen in Figure 2a, the load resistor representing the sample includes a capacitor that emulates the interaction of the electrodes with the sample. The electrodes form a capacitive equivalent (separate parallel metal plates) together with the sample. In the operational tests, only the conductive characteristics of the sample are considered.

2. Although the basic example outlines a single switch, in real applications, transistor arrays will be required to be able to handle the required high voltage levels. Figure 3 shows a basic schematic of the power converter, where transistor arrays are present at its output to generate positive and negative high-voltage pulses. The array structure is based on the MMC (Modular Multilevel Converter) topology [40]. The multiple switching signals to balance voltages across capacitors in the MMC structure require proper sequencing [41]. The logic operation and structure of the arrays are explained in the following section.

3. There are impedance variations in the sample being treated, and these are derived from its characteristics before and after being subjected to PEF. In the simulation, this change will be shown as a linear variation in resistance over a complete operating cycle.

4. The converter is based on a first stage similar to a Vienna rectifier, where operation is restricted to switching transistors according to the present AC half-cycle. The output of this configuration is coupled with a multilevel structure of diode–capacitor arrays (MBC—Multilevel Boost Converter) [42] to raise the voltage even higher than the Vienna configuration allows without causing significant stress on the switches in its configuration.

5. The selected controller is shown in Figure 3 and consists of a voltage-regulating control loop and a current-based switching control loop. A hysteresis current control structure is used to provide a near-unity power factor. In addition, low current distortion is achieved, and a small ripple tends to occur in the output voltage [43].

### Milk Conductivity Characterization

To validate the present proposal for a PEF-generating converter, it is necessary to use a characteristic load equivalent to that of milk. In general, milk is seen as a conductance in most of the literature. In this case, based on the ranges established in [44,45,46,47], two scenarios will be simulated. The first condition considers that the milk has been freshly extracted, and the second condition considers the change when the milk is treated. The dynamics established for the simulations will be shown by decreasing and increasing the load during the application of the PEF to the milk. These changes are considered because milk will flow continuously within the treatment chamber.

In the case of raw milk, a conductivity of 4680 μS/cm is reported in [44], but it tends to increase if the pH is modified due to the increase in lactic acid. This conductivity is consistent with [45], where it is mentioned that the maximum average values are between 4870 and 5300 μS/cm. The average of these conductivity data (5085 μS/cm) will be taken as the initial value (untreated sample) in the simulation tests. In the case of the treated sample, there is no particular conductivity data since this depends on the milk’s final conditions (whole, semi-skimmed, fat- and lactose-free, lactose-free). The average value of the samples reported in [47] will be used for whole milk, whose conductivity was obtained at a frequency similar to that at which the pulsed electric field (PEF) will be induced. To validate the good behavior of the converter, only the real part that corresponds to a value of 2626 μS/cm will be taken. The imaginary part arises from the capacitance formed by the distance between the electrodes and the sample to be treated.

In the next section, the proposed converter topology is presented, the allowed modes and their appearance conditions are analyzed, and the operating logic of the switching law is designed.

## 3. Proposed Power Converter and Control Law Design

The proposed converter is composed of two parts: the first for AC-DC voltage conversion and the second for high-voltage DC-DC pulsed output. The AC-DC conversion stage is based on a single-phase Vienna rectifier, enhanced with diode–capacitor cells to increase the voltage gain of the converter. Since the voltage amplitude required for PEF exceeds 10 kV [28], no commercially available transistors can directly control or generate such high-voltage pulses. Therefore, the high-voltage pulsed output stage is based on a Modular Multilevel Converter (MMC), modified to achieve the required voltage levels and short-duration pulses for PEF.

**Figure 4:**
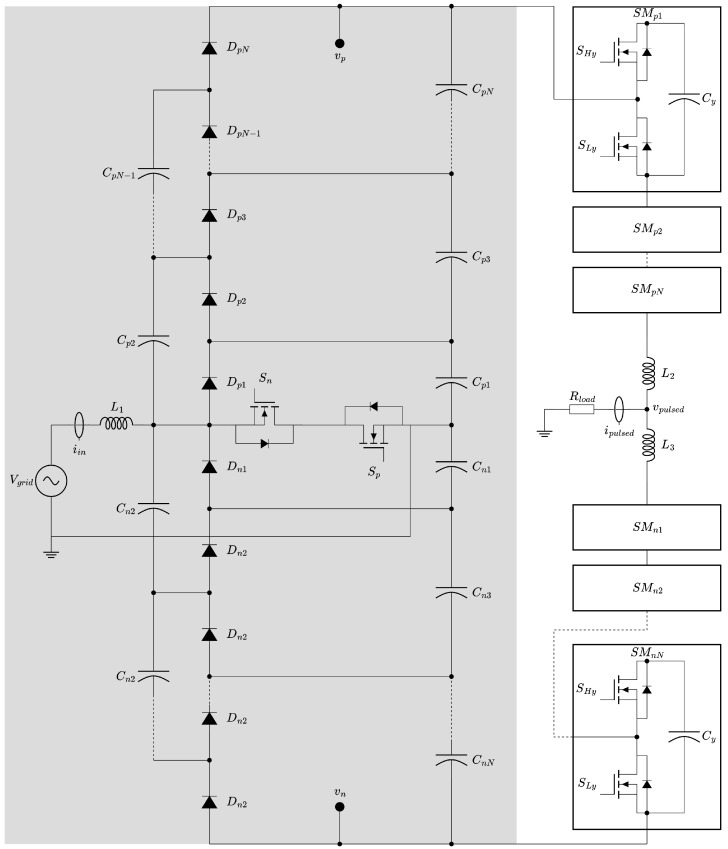
Proposed power converter schematic composed of the modified single-phase Vienna rectifier (shaded region) and the MMC-based high-voltage DC-DC pulsed output part.

As bipolar square wave pulses are preferred for PEF, an additional advantage of our proposed MMC-based converter is its ability to generate such waveforms, as shown in Figure 2b. Figure 4 presents a schematic of the proposed power converter, detailing both the AC-DC conversion and the high-voltage pulsed output stages. This converter also benefits from using the grid as its input source, eliminating the need for a separate AC-to-DC conversion stage to reach the required high voltage levels. As a result, the available power is limited only by the capacity of the grid.

Finally, with only two main stages to control, the proposed topology features simple operation. Table 3 lists the preliminary components selected for the proposed converter, from which their main characteristics are derived and used in the simulations described in the next section.

### Proposed Controller

The proposed power converter is composed of two parts, and the first part is responsible for generating the AC-DC conversion. The proposed controller operates similarly to a traditional Vienna rectifier, but with the added task of monitoring the high-voltage nodes after the diode–capacitor cells. This is achieved by measuring the $V_{p}$ and $V_{n}$ voltages, as shown in Figure 4, without adding significant complexity to the control scheme. Therefore, the proposed controller is based on the hysteresis-controlled Vienna rectifier presented in [48]. This control method offers a suitable solution for single-phase implementations, while state-space vector control can be used for three-phase systems to reduce total harmonic distortion [49,50]. As such, the AC-DC controller is well established in the literature and will not be discussed further.

Although the MMC has traditionally been applied in high-voltage DC transmission systems operating at megawatt levels [51,52], its characteristics also make it highly suitable for the proposed power converter. It enables control of high-voltage outputs using transistors with lower breakdown voltages than the source, thanks to the series arrangement of its submodules (SMs), as illustrated in Figure 4. To generate a bipolar pulse, the MMC must be supplied with both positive and negative voltages ($v_{p}$ and $v_{n}$ in our diagram) with the load $R_{load}$ connected between the neutral point and the midpoint of the MMC through the inductors $L_{2}$ and $L_{3}$. For the positive part of the pulse, the output voltage $V_{pulsed}$ is determined by

(1) $$\begin{array}{c} V_{pulsed}\left(t\right)=\sum_{y=1}^{SM_{pN}}S_{y}\left(t\right)V_{Cy}, \end{array}$$

and for the negative portion, it is determined by

(2) $$\begin{array}{c} V_{pulsed}\left(t\right)=\sum_{y=1}^{SM_{nN}}S_{y}\left(t\right)V_{Cy}, \end{array}$$

where $V_{Cy}$ is the capacitor voltage of the *y*th submodule, $SM_{pN}$ and $SM_{nN}$ are the positive and negative SM arrays, respectively, and $S_{i}\left(t\right)$ corresponds to the switching function of the *y*th SM:

(3) $$\begin{array}{c} S_{y}\left(t\right)=\left\{\begin{array}{c} 1,\mathrm{if}\;\mathrm{the}\;y\mathrm{th}\;\mathrm{submodule}\;\mathrm{is}\;\mathrm{inserted}\\ 0,\mathrm{if}\;\mathrm{the}\;y\mathrm{th}\;\mathrm{submodule}\;\mathrm{is}\;\mathrm{bypassed}. \end{array}\right. \end{array}$$

For an ideal balanced condition, all capacitor voltages $V_{C}$ will be the same; therefore, for a bipolar pulse, we can obtain the following:

(4) $$\begin{array}{c} V_{pulsed}\left(t\right)=\left\{\begin{array}{c} +v_{p},\;\mathrm{if}\;k_{p}>k_{n}\\ 0,\;\mathrm{if}\;k_{p}=k_{n}\\ -v_{n},\;\mathrm{if}\;k_{n}>k_{p}, \end{array}\right. \end{array}$$

where $k_{p}$ is the number of inserted SMs in the positive arm, and $k_{n}$ is the number of inserted SMs in the negative arm.

Therefore, it is necessary to balance the voltages of the capacitors $v_{C}$ in the SMs. Multiple techniques have been analyzed. In [53], a classification network control implemented in an FPGA is presented, or in [54], a decentralized voltage balancing algorithm is presented. Those techniques are based on the modulation of a sine wave for AC interconnection. Hence, as we want to control a pulse for the PEF, one possible way to achieve a balancing condition for the capacitors is by slightly varying the turn-on sequence of the SMs. This is achieved in the controller by randomizing a delay for each SM turn-on condition. This can be defined as follows:

(5) $$\begin{array}{c} \tau_{i}=\alpha_{i}\cdot T, \end{array}$$

where $\alpha_{i}$ is the random value uniformly distributed in $\left[0,\tau_{\max}\right]$, *T* is the switching period, and $\tau_{\max}$ is the maximum allowable delay. Adding this random delay to Equation (4) results in the following:

(6) $$\begin{array}{c} V_{pulsed}\left(t\right)=\sum_{y=1}^{SM_{pN}}S_{y}\left(t-\tau_{i}\right)V_{Cy} \end{array}$$

## 4. Simulation Results

Based on the component selection described in the previous section, this section details the simulations carried out to validate the proposed power converter. Taking into account the available transistors for both the Vienna rectifier and the MMC submodules (SMs), the number of diode–capacitor cells and SMs was selected to achieve the target voltage amplitude of 10 kV required for PEF. This resulted in five diode–capacitor cells for both the positive and negative sections of the Vienna rectifier and N=6 SMs for each of the positive and negative arms. This configuration provides a sufficient voltage margin to accommodate transient voltage spikes during transistor switching.

**Figure 5:**
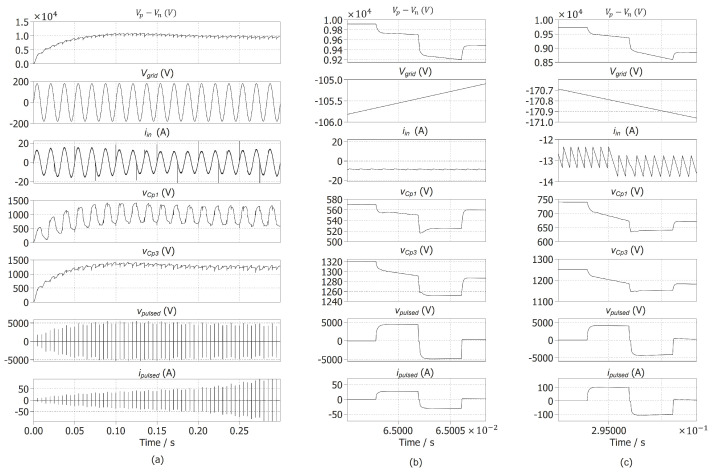
Proposed power converter simulation results (**a**) for 300 ms, (**b**) zoomed-out version when reaching 10 kV ($v_{p}-v_{n}$), and (**c**) zoomed-out version at the end of the impedance change.

Validations of the proposed power converter were conducted using simulations performed in the sponsored PLECS software. The load impedance was modeled as a linear change from 196 Ohms to 38 Ohms, as discussed in Section Milk Conductivity Characterization. This change occurs over the full simulation time. Figure 5a shows that the converter successfully reaches the desired 10 kV voltage amplitude ($v_{p}-v_{n}$) in under 100 ms and adapts effectively to the varying output impedance. A zoomed-out view is presented in Figure 5b, illustrating the bipolar output pulse generated by the MMC-based converter ($v_{pulsed}$), which achieves a 10 kV peak amplitude with a 4 µS duration, as required for PEF.

**Figure 6:**
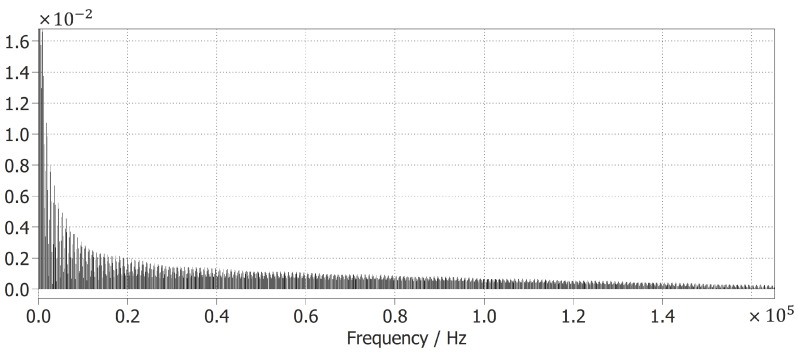
Proposed power converter hysteresis command controller FFT.

From Figure 5a, we can also see that the input current $i_{in}$ is in phase with the grid voltage, so a near-power factor of 1 can be achieved with the converter. In addition, the total harmonic distortion of the converter was calculated, taking the input current $i_{in}$ as described in [55]. The converter achieves a THD of 1.74%, which is well below the IEEE Std-519 [55]. Furthermore, Figure 6 shows an FFT calculation of the switching commands for $S_{n}$ and $S_{p}$, symbolizing that the frequency range is between 60 Hz and 20 kHz, decreasing linearly past this range. Therefore, the hysteresis controller is validated and can be implemented physically, and this range additionally reduces the stress in the transistors.

**Figure 7:**
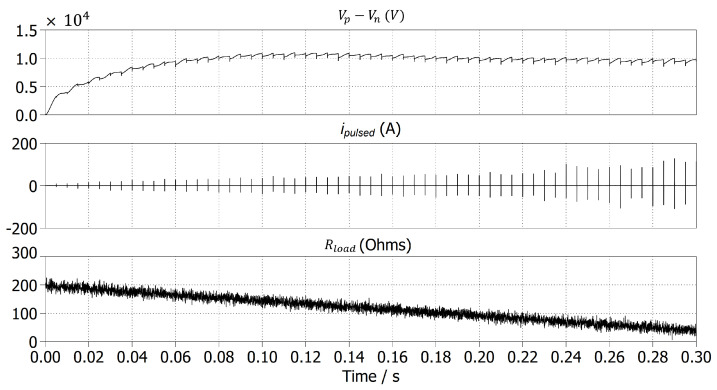
Proposed power converter simulation results with added Gaussian noise at $R_{load}$.

Additionally, as seen in the simulations of Figure 5b,c, by measuring the changes in $i_{pulsed}$, we can observe the change in impedance, representing the electrical conductivity of the processed sample. For this simulation, we contemplated a duration of 300 ms for the impedance change, as observed from Figure 5a. Figure 7 illustrates the same impedance change with the addition of Gaussian noise, with a standard deviation value of 10, simulating possible fluctuations that could arise during milk processing, validating the voltage regulation of the proposed converter and the capability of the MMC-based switch to operate under such random changes at the output.

## 5. Discussion

Our study focuses on generating a device to treat fluid milk in a non-thermal way. Therefore, we are developing a proof of concept for a power converter design intended for PEF food processing. We chose this food product because it is a vital nutritional source for consumers, as well as a complex food product that includes all the macronutrients and micronutrients [1,56]. Therefore, the present challenge is to develop a suitable technology for the treatment of milk using non-thermal methods that have been developed, taking into account the needs of consumers such as food safety (considering microbiological safety) and a minimal change in the nutritional and sensory properties of treated milk [1,2,57].

The device that we propose works with pulsed electric field (PEF) electroporation, which is based on using high-frequency, high-voltage pulsations generally below 50 KVcm^−1^ for a short time duration (generally µs to ms). Therefore, this technology operates by inactivating or inhibiting microorganisms based on the disturbance and perforation of the membrane of cells by the formation of pores, as well as the rupture of protein channels in the membrane [8,9,10].

This work presents the operation and design of the proposal through simulations without considering the modeling of the treatment chamber. Treatment chamber modeling has not been considered because precise physical data are not yet available. It is possible to model the chamber as an RC array, as mentioned in [47], where the milk’s conductivity is specified together with the capacitance produced by the separation of the electrodes within the chamber. This is shown circuitally as a parallel arrangement between a capacitance and a series of capacitances and resistors. However, the electrode/milk interface cannot be modeled as an ideal capacitor due to variations in sample density and the distance between electrodes. In the same reference, variations from 1.41 to 2.03 nF are mentioned. Only the milk’s conductance is used in the simulations because it is the ideal parameter to verify the power demand in the proposed topology. In this case, validation is performed using a characteristic load impedance that emulates the change in resistivity from an untreated sample to that of a sample treated with PEF.

The device we propose includes a scalable power converter designed to process larger volumes and faster throughput of dairy products as required for PEF applications. The Vienna rectifier can be readily adapted to a three-phase system, and the MMC offers a modular solution by allowing the addition of more submodules (SMs) in series or parallel.

Moreover, given that designing a regulated high-voltage power supply can be both expensive and complex, this work proposes leveraging the Vienna rectifier architecture as a foundation. This approach offers the advantage of converting the AC input directly into high-voltage DC, with the power capacity depending on the selected components. To further increase the voltage gain, a stage based on capacitor charging arrays (a multilevel converter) is added to the rectifier structure.

The resulting high-voltage output is then coupled with cascaded switch arrays to generate bipolar pulses. These switches are triggered at precise intervals to prevent the appearance of excessive voltage levels. The basic control loop is implemented using hysteresis current control, ensuring that the input current remains in phase with the voltage. The timing of the processing cycle was designed based on a linear variation in the sample’s impedance. Simulation results confirm that the converter maintains the desired output levels and that the switches experience low stress, even under varying impedance conditions.

We believe that this device holds potential for industrial applications, provided it can be demonstrated to produce safe, nutritious, and high-quality products. Therefore, validating its functionality through accurate kinetic studies is essential [57]. One limitation of this study is that it does not include the modeling of the treatment chamber.

Future work will focus on evaluating the microbial safety of the products processed using this equipment, as well as assessing their sensory and nutritional qualities. Additionally, we plan to conduct shelf-life studies.

## 6. Conclusions

In this research, we performed a proof of concept for the PEF method using a power converter design to be used for raw milk processing. As stated, the PEF method generates a high-voltage, high-frequency pulsating electric field that is applied to the food for a short duration, typically measured in milliseconds, and in this case, we performed the simulation using the electrical characteristics of raw milk and its change to processed milk.

This work highlights the following:

1. An operation and design of the proposed PEF converter through simulation.
2. This device and its proposed controller produce high output voltage and adaptive bipolar pulse generation, using a Vienna rectifier and Modular Multilevel Converter.
3. The system is validated using characteristic load impedance that emulates the change in resistivity from an untreated milk sample to one treated using PEF.

Finally, we can establish that the results obtained under the proposed conditions are sufficiently precise to demonstrate the feasibility of the proposed design of the PEF for raw milk samples.

## Figures and Tables

**Table 1:** Heat treatment in the milk industry. (The check mark indicates which physical characteristic is affected.) [5,6,7].

Heat Treatment	Temperature (°C)	Time	Main Effects	Drinkable	Odor (Cooked Milk, Caramelization, Sulfurous Notes)	Flavor (Cooked Milk, Caramelization, Sulfurous Notes)	Color	Texture (Viscosity)
Thermalization	57–68	5 s to 30 min	Mildest treatment. Not suitable for drinking milk, but extends raw milk shelf life before further processing (e.g., cheese).		✓	✓		
Pasteurization (LTLT)	63–65	30 min	Traditional pasteurization. Produces drinkable milk.	✓	✓	✓	✓	
Pasteurization (HTST)	72–80	15–30 s	Standard method for drinking milk. Kills pathogens, limited protein denaturation.	✓	✓	✓	✓	
High Pasteurization/ Yogurt	90–95	3–5 min	Used mainly in yogurt production. Not typical for drinking milk due to flavor changes.	✓	✓	✓	✓	✓
Extended Shelf Life (ESL)	125–140	1–10 s	Prolongs milk shelf life under refrigeration. Suitable for drinking milk if kept cold.	✓	✓	✓	✓	✓
Ultra-High Temperature (UHT)	135–150	1–10 s	Enables ambient shelf life. Standard for shelf-stable drinking milk.	✓	✓	✓	✓	✓
In-Container Sterilization	110–125	5 min or 10–20 min	Harshest treatment. Ambient-stable drinking milk, but with significant sensory changes (cooked notes, color darkening, thicker texture).	✓	✓	✓	✓	✓

**Table 2:** Summary of key features for PEF generators.

	[32]	[33]	[34]	[35]	[36]	[37]	[38]	[39]	[17,18]	[22,31]	Proposed
Number of switches	2 (at input)	5 (per stage)	2 (at input), 2 (per MMC module)	5 (IGBT and thyristors)	2 (at input), 14 (series MMC)	9 (all structure)	1 (per VBM—Voltage Boost Module), 2 (per MMC module)	2 (at input)	1 and 2, respectively	8 and 12, respectively	2 (at input), 2 (per MMC module)
Power supply	HVDC (DC source)	LVDC (DC source)	LVDC (DC source)	HVDC (battery bank)	LVDC (DC source)	LVDC (DC source)	LVDC (DC source)	LVDC (DC source)	HVDC	HVDC (several sources)	LVAC (voltage grid)
Output level	500 V	20 kV (peak to peak)	20 kV (peak to peak)	10 kV	10 kV	500 V	10 kV	1 kV	2.5 kV and 4.5 kV, respectively	Input source-dependent	10 kV (peak to peak)
Switch stress	High	High	High (input), Low (output MMC)	High (input), High (output filter)	High (input), Low (output MMC)	Low	Low	Low	High	High	Low
Waveform type	Unipolar pulse	Multiple waveforms	Bipolar pulse	Unipolar pulse	Multiple waveforms	Unipolar pulse	Unipolar pulse	Unipolar pulse	Unipolar pulse	Bipolar pulse	Bipolar pulse
Boost structure	DC-DC Boost	MARX circuit	MBC (Multilevel Boost Converter)	DC-DC Boost	Dual Boost converter	Transformer ratio	Multiple conventional DC-DC Boost with coupled Transformer	Half bridge with coupled Transformer	Without Boost structure	Without Boost structure	MBC (Multilevel Boost Converter)
Pulse structure	RLC circuit (Blumlein)	H-Bridge Transistor arrays	MMC half-bridge modules	Capacitor discharge by thyristor	Series Modular Multilevel Converter (SMMC)	Secondary Transformer induction	MMC half-bridge modules	Charge–discharge capacitor with diode bridge structures	Capacitor discharge by switches	H-bridge transistor arrays	MMC modules
Pulse duration	nS	μS	μS	mS	μS	mS	μS	mS	nS, μS	μS	μS

**Table 3:** Components used for simulations.

Item	Model	Description
$S_{n}$, $S_{p}$	2× GC2M0160120D	SiC transistor 1.2 kV 160 mOhm
$SH_{y}$, $SL_{y}$	2× GC2M0160120D	SiC transistor 1.2 kV 160 mOhm
Gate driver	Si8233AD	High-side/low-side 5 kV isolated driver
Dpx and Dnx	CI02S120C3	1.2 kV SiC diode 10 A
Measurement	ADE9113	Isolated voltage and current amplifier
Cpx and Cnx	MPP105J2000D01	Film capacitor 1 uF 2 kV
Cy	MPC104J1200D01	Film capacitor 100 nF 1.2 kV
$L_{1}$	EEF432130C	170 uH 35 A inductor

## Data Availability

The original contributions presented in this study are included in the article. Further inquiries can be directed to the corresponding authors.
